# The Public Health Impact of Coronavirus Disease on Human Trafficking

**DOI:** 10.3389/fpubh.2020.561184

**Published:** 2020-10-29

**Authors:** Jordan Greenbaum, Hanni Stoklosa, Laura Murphy

**Affiliations:** ^1^Institute on Healthcare and Human Trafficking at the Stephanie V. Blank Center for Safe and Healthy Children at Children's Healthcare of Atlanta, Atlanta, GA, United States; ^2^HEAL Trafficking (Health, Education, Advocacy, Linkage), Los Angeles, CA, United States; ^3^Department of Emergency Medicine, Brigham and Women's Hospital, Harvard University, Boston, MA, United States; ^4^Helena Kennedy Center for International Justice, Sheffield Hallam University, Sheffield, United Kingdom

**Keywords:** coronavirus disease, COVID-19, human trafficking, health impact, prevention, public health, sex trafficking, labor trafficking

## Abstract

The global pandemic of severe acute respiratory syndrome coronavirus 2 exacerbates major risk factors for global human trafficking. Social isolation of families and severe economic distress amplify the risk of interpersonal violence, unemployment and homelessness, as well as increased internet use by under-supervised children. Aggravating the situation are overwhelmed health systems, severe limitations in activities of social service organizations, and decreased contact of healthcare professionals with children. Healthcare professionals have a duty to be alert to possible indicators of trafficking, and aware of available victim resources which can be offered to at-risk patients. Healthcare facilities should take steps to increase public awareness of trafficking and community resources.

## Introduction

Human trafficking is a major global public health problem (1, 2). In all likelihood, the prevalence of exploitation will increase as a result of the severe acute respiratory syndrome coronavirus 2 (SARS-CoV-2) pandemic (3–6). Trafficking involves the exploitation of others using force, fraud, coercion, or abuse of power, principally in the contexts of commercial sex and forced labor (7). The impact of human trafficking ranges from individual health consequences (e.g., traumatic injury, infections, unwanted pregnancy, malnutrition, exposure to toxins, post-traumatic stress disorder, and depression) to family strife and division, community discrimination, business expectations for docile and unpaid labor, and societal norms of cheap consumer goods.

The recent global outbreak of SARS-CoV-2 has led to major social changes severely limiting social interaction and restricting movement of individuals and populations. While research on the specific impacts of COVID-19 has yet to emerge, the current state of knowledge provides some insight into the ways such a global crisis will likely exacerbate vulnerabilities to trafficking (5, 6). Social distancing and closures of schools and businesses have decreased in-person contact and expanded online communication. Dramatic changes in the economy have also significantly increased unemployment, poverty, and homelessness (8). The changes have the potential to markedly increase the risk of human trafficking both during and after the pandemic. Notably, baseline risk for trafficking is not equally distributed in society. People of color, gender minorities, migrants, those with a history of abuse, and the working poor are more likely to experience trafficking (9–14). The pandemic's impacts will likely magnify these risks among already marginalized populations. COVID-related trafficking risks may be grouped into three interrelated categories: family life, economic distress, and limits in external professional assistance. Intensification of family strains due to quarantine may be exacerbated by both economic strain and limited access to services, all combining to produced new and heightened vulnerabilities, especially for those marginalized populations who are unequally affected by both COVID-19 and human trafficking. It is incumbent upon HCPs to be aware of these heightened risk factors during pandemics.

## Family Life

1. **Rise in intimate partner violence (IPV) and child maltreatment**. Family violence, including IPV and child abuse are risk factors for human trafficking (15, 16). Given the mandated restrictions in movement seen in many countries as part of the coronavirus disease (COVID-19) mitigation effort, as well as closures of alternative safe housing, and increased financial pressures, families struggling with violence and interpersonal dysfunction are forced to spend increasing amounts of time together, which may exacerbate tensions, Reports related to IPV and child sexual exploitation increased in multiple countries during the early period of the Covid pandemic (17, 18). Increased financial strains may result in family members exploiting each other into forced labor. Overcrowding within a residence, and/or the presence of a sexual offender may render children in the home vulnerable to sexual abuse or exploitation. Maltreated children are less likely to come to the attention of mandated reporters now that schools are closed (19). This allows abuse to go unchecked and potentially drives a child to run away from home, rendering them at an even higher risk of exploitation.
2. **Increased use of internet by children**. With school closures many children turn to social media and other online activities to fill their time. For those who engage in risky online behaviors such as acting aggressively toward others, or sharing personal information with people met online (20), and who have risk factors associated with offline sexual abuse may experience increased risk of online sexual exploitation, especially if the added time on the internet occurs in the context of limited supervision (21). The allure of fraudulent online job ads can also increase risk of labor trafficking for children, youth, and adults who lack safe job searching skills.

## Economic Distress

1. **Labor exploitation/trafficking**. Globally, economic stress could increase cross-border migration in search of work, which can put people at risk of exploitation (9, 22). Simultaneously, border closure to prevent the spread of infection could limit workers' options to migrate for safer work conditions outside their home countries or increase the cost associated with migration (23). In these situations as well as others involving increased economic crisis due to COVID-19, desperate adults and youth may be forced to accept exploitative, coercive, unpaid, or inescapable work conditions (5, 6). Caregivers may allow children to engage in hazardous child labor such as work with dangerous machinery, or work in an unhealthy environment. Children and youth may be induced to engage in illicit activities to earn money, such as selling or transporting drugs.
2. **Sexual exploitation/trafficking**. Caregivers in financially fragile positions at baseline may resort to sexual exploitation of children to pay for food or other necessities (24). Adolescents may decide to assist the family in obtaining money by selling sex. Adults involved in consensual commercial sex may find it more difficult with COVID-19 restrictions to solicit clients, forcing them to engage in riskier behaviors and to accept clients who might present greater danger of abuse, rape, or trafficking (25).
3. **Traffickers**. In addition to the increased vulnerability described above, economic strain could encourage people to engage in illicit activity, including compelling or coercing others into unpaid labor or forced sex work (6, 24, 26). Addressing economic strain is as relevant to preventing victimization as it is to preventing perpetration.

## Limits in Assistance from Professionals

1. **Overwhelmed health systems**. The surge in hospital and emergency department admissions related to COVID-19 has overwhelmed health systems in many countries. Major concerns about infection exposure, PPE and ventilator shortages, and treatment of severely ill patients take priority in the attention of healthcare professionals (HCP) and administrators. Understandably, other urgent situations including human trafficking may be overlooked and the opportunity for offering resources to exploited persons missed. HCPs may assume there is no time to screen for trafficking and exploitation or to spend time counseling about worker rights, community referrals, and resources.
2. **Overwhelmed social service agencies**. As social service agencies struggle with cuts to funding and keeping clients and staff safe, typical face-to-face contact and service provision for high risk families may be limited (4). Organizations providing services to trafficked persons, immigrants/refugees, and homeless/runaway youth and adults will need to shift outreach techniques to identify and serve those in need.
3. **Under-staffed law enforcement agencies**. Economic desperation, homelessness hunger, and anti-immigrant bigotry may lead to marked increases in crime and general social unrest. Over-stretched law enforcement staff may shift their focus away from trafficking investigations.

## Discussion: What Can HCPs Do?

Trafficking is a public health issue that affects people of all ages, races, genders, nationalities, and sexualities. While trafficking and other forms of violence occur regardless of pandemics or natural disasters, it is critical in moments of heightened risk that HCPs equip themselves to be particularly vigilant and prepared to assist survivors. The following are recommendations that HCPs can implement to address the overlapping heightened trafficking risk factors related to familial life, economic distress, and HCP capacity. These actions are designed to increase the likelihood of identifying individuals at risk of trafficking and providing them appropriate care, regardless of the underlying exacerbating circumstances, which will often be obscured to the practitioner.

1. While HCPs may have very little time to spend with patients in person, especially those without concerns of SARS-CoV-2 infection, even emergency department staff can offer at-risk patients written resources related to worker rights, IPV, national human trafficking hotlines, and/or immigrant/refugee services (27). If delivering healthcare via telemedicine the provider can type in the links in the “chat” box. Such resources can be downloaded from HEAL Trafficking (https://healtrafficking.org/patient-resources/, and https://healtrafficking.org/covid-19-resources-2/).
2. When conducting telemedicine evaluations, HCPs should be observant of conditions in the patient's environment. If such a visit occurs in the home of a trafficked/exploited person, possible indicators of exploitation may be evident, such as apparent bullying or violence occurring in the home environment, other suspicious activity in the background, or the presence of a domineering companion who wants to speak for the patient.
3. Healthcare facilities should display posters on human trafficking, IPV and other forms of violence, including contact information for resources and other assistance for both patients and providers. These posters should avoid using sensational images that reinforce stereotypical images of white cis-gendered female sex trafficking victims, and rather capture a diversity of lived experience, in order to improve outreach to the most affected communities.
4. HCPs may collaborate with community providers serving vulnerable populations and, with the patient's permission, refer patients for virtual or in-person services. Lists of national online resources are also a potential source of assistance to vulnerable patient populations.
5. Encourage HCPs to identify community and government agencies providing emergency support (e.g., food and basic supplies) and make a list of such service providers easily accessible to patients who travel to health facilities or engage in telehealth sessions.
6. Encourage health professional organizations to advocate for labor rights and enforcement of labor laws, especially those related to minimum wage, work hour limits, safety requirements, and healthcare benefits for workers.
7. Since there is significant overlap in risk factors for online and offline sexual abuse/exploitation, as well as for online and offline labor exploitation, research suggests that HCPs use established strategies for offline abuse prevention to help reduce the risk of online exploitation. For example, they may counsel pediatric patients and caregivers about healthy and unhealthy relationships, the importance of consent, respect for others, safety planning, and safe job search strategies (28).
8. Talk to caregivers about joining online support groups or having their children join safe, supportive, well-monitored peer groups.
9. If an HCP suspects exploitation/trafficking, they should report concerns to authorities ***IF*** (1) the patient appears to be in imminent danger (call emergency services), (2) the HCP has a mandatory reporting obligation, or (3) the report is not mandatory but the patient requests law enforcement involvement. All HCPs should be aware that reporting can lead to negative consequences including (but not limited to) criminalization of victims, immigration proceedings, and violent repercussions, so it is imperative that patient requests regarding law enforcement engagement be respected when possible, given mandatory reporting laws. In addition, if the decision is made to involve law enforcement, every effort should be made to engage the members of law enforcement most informed about the complexities of trafficking.

For information on how HCPs can assess for trafficking and connect trafficked patients to resources, see **HEAL Trafficking (resources for health professionals):** **www.HEALtrafficking.org**. Health professionals may connect trafficked persons with the **National Human Trafficking Resource Center hotline: 1-888-373-7888 (SMS “BEFREE”) 24/7**.

## Data Availability Statement

The original contributions presented in the study are included in the article/supplementary material, further inquiries can be directed to the corresponding author/s.

## Author Contributions

All authors listed have made a substantial, direct and intellectual contribution to the work, and approved it for publication.

## Conflict of Interest

The authors declare that the research was conducted in the absence of any commercial or financial relationships that could be construed as a potential conflict of interest.
